# Self-expanding metal stents in malignant colonic obstruction: experiences from Sweden

**DOI:** 10.1186/1756-0500-4-274

**Published:** 2011-07-30

**Authors:** Mattias Lepsenyi, Stefan Santen, Ingvar Syk, Jörgen Nielsen, Artur Nemeth, Ervin Toth, Henrik Thorlacius

**Affiliations:** 1 Department of Surgery, Skane University Hospital Malmö, Lund University, S-20502 Malmö, Sweden; 2 Department of Emergency Medicine, Skane University Hospital Malmö, Lund University, S-20502 Malmö, Sweden

## Abstract

**Background**

Acute surgery in the management of malignant colonic obstruction is associated with high morbidity and mortality. The use of self-expanding metal stents (SEMS) is an alternative method of decompressing colonic obstruction. SEMS may allow time to optimize the patient and to perform preoperative staging, converting acute surgery into elective. SEMS is also proposed as palliative treatment in patients with contraindications to open surgery. Aim: To review our experience of SEMS focusing on clinical outcome and complications. The method used was a review of 75 consecutive trials at SEMS on 71 patients based on stent-protocols and patient charts.

**Findings**

SEMS was used for palliation in 64 (85%) cases and as a bridge to surgery in 11 (15%) cases. The majority of obstructions, 53 (71%) cases, were located in the recto-sigmoid. Technical success was achieved in 65 (87%) cases and clinical decompression was achieved in 60 (80%) cases. Reasons for technical failure were inability to cannulate the stricture in 5 (7%) cases and suboptimal SEMS placement in 3 (4%) cases. Complications included 4 (5%) procedure-related bowel perforations of which 2 (3%) patients died in junction to post operative complications. Three cases of bleeding after SEMS occurred, none of which needed invasive treatment. Five of the SEMS occluded. Two cases of stent erosion were diagnosed at the time of surgery. Average survival after palliative SEMS treatment was 6 months.

**Conclusion**

Our results correspond well to previously published data and we conclude that SEMS is a relatively safe and effective method of treating malignant colonic obstruction although the risk of SEMS-related perforations has to be taken into account.

## Background

Acute or pending colonic obstruction, on the basis of colorectal malignancy, is a condition traditionally treated with surgery with a single or a multi-stage operative procedure. Acute surgery with colonic resection and primary anastomosis with or without a diverting stoma is the most common surgical approach in relatively healthy patients. The multi-stage approach includes initial decompression of the obstruction with a temporary colostomy and a later colonic resection with reversal of the stoma if possible. This method is mainly considered in patients with deteriorated general condition, serious co-morbidity or in cases with advanced tumors. Regardless of the technique used, surgical procedures are associated with a high morbidity and mortality rate [1]. The use of self-expanding metal stents (SEMS) for decompression of colonic obstructions was first described by Spinelli et al [2] in 1992 as a palliative treatment. In 1994, Tejero et al [3] published a preliminary report on the use of SEMS as a temporary treatment as a bridge to surgery, relieving the patient of the obstruction without the need for emergency bowel surgery. This allows time for optimization of the patient's physiological parameters, completion of preoperative staging and conversion of an acute surgical intervention into an elective procedure. The method has since then been taken into clinical practice internationally and in Sweden, both for palliative treatment and as a bridge to surgery [4-18].

The aim of this study was to review our series of cases with regards to technical and clinical outcome and complications.

## Methods

Patient documentation was reviewed retrospectively in 75 consecutive attempts at placing SEMS on 71 patients (47 men, 24 women). The procedures were performed during a six-year period. The mean age of the patients was 74 years. In 64 (85%) cases, the indication was palliative treatment. In 11 (15%) cases, the intention was bridge to surgery, with the intention to perform elective surgery after patient optimization and possibly further diagnostic procedures.

The diagnosis of colonic obstruction was based on clinical signs, such as abdominal distension, constipation and supportive radiological work-up. Computed tomography (CT) was performed in 64 (90%) patients, colonoscopy in 32 (45%), water soluble contrast enema (CE) in 25 (35%), plain abdominal x-ray in 23 (32%) and rectoscopy in nine (13%) patients.

All patients were conscious during SEMS deployment and had the opportunity to receive conscious sedation in the form of midazolam hydrochloride (F. Hoffmann-La Roche Ltd. Basel, Switzerland) and analgesia with ketobemidon chloride (Pfizer Inc. New York, USA) intravenously. Combined endoscopic and radiological techniques were used, where the distal end of the obstruction was located endoscopically. After placing a guide-wire through the obstruction, an endoscopic filling catheter was inserted over the guide-wire. Water-soluble contrast medium was injected to visualize the proximal end of the tumor. After reinserting the guide-wire, the catheter was removed and the stent was inserted and deployed. Stent expansion was confirmed with a radiological control image immediately after deployment. In three (4%) cases, there was a need to place a second stent coaxially in the first one to get an adequate coverage of the obstruction. Boston Scientific Wall-stent and Wall-Flex stents (Boston Scientific/Microvasive, Natick, MA, USA) were used; 22 nine-cm Wall-Flex, 28 12-cm Wall-Flex, two six-cm Wall-Stents, 15 nine-cm Wall-Stents and one 12-cm Wall-Stent.

This research is in compliance with the Helsinki declaration and has been approved by the regional research ethics board at Lund University, Sweden.

## Findings

The sigmoid and recto-sigmoid junctions were the most common locations of the obstruction, representing 31 (44%) and 15 (21%) cases respectively. Five (7%) lesions were found in the rectum, nine (13%) obstructions were located at the splenic flexure and eleven (15%) obstructions were located proximal to this. The median stricture length was four cm (range 2-10 cm). Technical success was visually defined as adequate stent placement overlapping the proximal and distal end of the stricture by two cm with a clear-cut waist line of the stent on a plain radiograph. This was achieved in 65 (87%) attempts. Notably, faecal flow through the stent was registered in 30 (46%) cases at the end of a technically successful procedure, indicating immediate functional decompression. Clinical decompression was defined as stool and gas passage within 48 hours after stent deployment, which was achieved in 60 of 75 cases, resulting in a clinical success rate of 80% success rate of all attempts made or 92% of the technically successful cases. In two additional cases, clinical decompression was achieved within one week after prolonged intestinal paralysis. Reasons for technical failure were failure to cannulise the stricture with the guide-wire, which made stenting impossible in six (8%) cases and incorrect stent placement in three (4%) cases. In one case, the tumor was not sufficiently stenotic and the SEMS migrated distally immediately after placement. It was removed during the same procedure. The average time for the entire procedure was 68 min (range 13-171 min).

In the eleven patients stented as bridge to surgery, operations were all open laparotomies performed one to 31 days after stent placement with one outlier at 90 days post-SEMS treatment due to administration of neoadjuvant chemotherapy. In eight patients, a one stage curative operation with primary anastomosis was performed. Two patients underwent a Hartmann's procedure with a left colon end stoma and one patient with advanced malignancy had a diverting stoma as the only treatment.

In terms of clinical complications, we observed four cases (5%) of supposed stent procedure-related bowel perforation. In two cases, perforation of the caecum was detected shortly after stent placement in colon. These patients underwent acute open surgery but both died of complications one and 16 days postoperatively. In the third case, CT shortly after the procedure showed a covered perforation in the mesentery adjacent to the stent. This patient did not develop peritonitis and was treated conservatively with successful outcome. A forth case of perforation occurred, in which the catheter and guide-wire perforated the bowel wall in a necrotic tumor area and contrast fluid was detected in the abdominal cavity upon injection. This patient was not stented and underwent emergency surgery and tumor resection without further complications. Stent erosion through the bowel wall, presumably due to mechanical forces, was detected in two patients; one in junction to surgery and one described by the pathologist. These two patients were operated on eight and 23 days after stent placement. Both patients had a Wall-Flex 12 cm stent. None of them had any visible leakage of bowel content but merely a localized inflammatory reaction with the mesentery covering the site of erosion. Stent occlusion occurred in five cases (7%) after 75, 79, 95, 130 and 195 days. One case was decompressed by restenting and one by flushing the occlusion via colonoscopy. One underwent re-stenting but never decompressed clinically. Three eventually had open surgery. In four of the five stent occlusions, a Wall-Flex 12 cm stent was used. In three cases (4%), a macroscopic bleeding was observed after the procedure, presumably induced by the stent deployment. All cases of SEMS-related bleeding seized spontaneously. One case of stent migration occurred in a patient who had chemotherapy after SEMS treatment. In this case, the stent was washed out with the stool after nearly three months and just prior to planned surgery.

The average survival time after palliative stenting was six months with an average follow-up time of 18 months. Of the 11 patients undergoing stenting as a bridge to surgery, two patients with serious co-morbidity died shortly after surgery. One went abroad without any possibility to follow up. The other eight cases successfully underwent elective surgery without complications and were all still alive at the time of data collection.

## Discussion

Primary colorectal carcinoma is one of the most common causes of colonic obstruction. Acute obstructive symptoms are the first presentation of illness in seven to 30% of the patients [19-21]. An acute surgical procedure has traditionally been used to ameliorate the obstruction. In general, a two or three stage operative strategy has been used where decompression are achieved by colostomy combined with tumor resection primarily or as a second procedure and stoma reversal in a second or third operation [22]. Stoma reversal is often abandoned in cases of advanced malignancy or serious co-morbidity [21,23]. Due to high morbidity and mortality rates [20], a strategy based on primary resection and anastomosis has been advocated. This strategy is now accepted as the standard surgical treatment in most cases [24-26] although there is no evidence that one therapeutic approach is superior to the other [1]. In cases with advanced cancer, a palliative and permanent end colostomy for bowel decompression has been the treatment of choice. However, this procedure is also associated with high morbidity and mortality rates. Considering the high risks involved in emergency surgery, a less traumatic way of decompressing the obstruction is of great interest.

During the last 15 to 20 years, SEMS have become the major endoscopic method to relieve malignant colonic obstruction, both as a palliative measure and as a temporary treatment before elective surgery [4-18]. SEMS as a bridge to surgery has also been compared to acute operation with favorable clinical outcome [10,14-18,27,28]. Complication rates after SEMS compared to surgical intervention are generally lower and of a less serious nature in most studies. An exception is the Dutch multicenter study, in which an unexpected high rate of perforations led to early closure [29]. SEMS as bridge to surgery allows a higher frequency of primary anastomosis when compared to emergency surgery and there are no significant differences in survival between the groups [30]. SEMS used as a palliative treatment are generally patent until the death of patients due to underlying malignancy. Notably, there is a very low rate of mortality related to SEMS complications in most studies [17]. Quality of life is also maintained at an acceptable level in patients treated with palliative SEMS [31]. Another advantage of palliative SEMS is that chemotherapy treatment can be administered earlier than after major surgery and in some cases allowing downgrading of the tumor for later liver surgery of metastasis [15]. As a conclusion, previous studies have shown that SEMS have the potential of reducing morbidity and mortality related to the management of acute malignant colonic obstruction if performed in a safe and effective way.

The present study focuses on procedural and clinical results, of SEMS treatment in our hands. Our technical success rate of 87% correlates well with previous findings, which frequently range between 66-100% in the literature. Considering that this study comprises all colonic SEMS undertaken in our centre and therefore may include a learning curve component for the endoscopists, the results are acceptable. A clinical success rate of 80% is also in accordance with other studies with a clinical success rate ranging between 46-100% [32]. The majority of obstructions were located in the recto-sigmoid area representing 71% of the cases. This is also in line with previous studies showing a predominance of left side colonic lesions around 70% [33]. In general, complications after SEMS are reported to be relatively few. In our study, the most serious complication was bowel perforation, which occurred in four (6%) SEMS cases. Two of these patients died due to complications of the subsequent emergency surgery. It is interesting to note that the level of serious morbidity has been reported to be around 4-5% of the cases according to a review by Watt et al [32]. In two other cases, stent erosion through the bowel wall was detected at the time of surgery. In neither case did the erosion lead to any significant leakage or peritonitis. Other less serious complications included minor bleeding, presumably from the SEMS site in three (4%) cases and stent occlusion, which occurred in five (7%) cases.

## Conclusion

This study shows that SEMS is a reasonably safe and effective procedure for treating obstructing colorectal carcinoma. The method has relatively few serious complications, although two perforations with lethal consequence are significant. A limitation of this study is its retrospective nature with inherent confounding factors. Further prospective randomized studies comparing SEMS with emergency surgery would be of great benefit.

## Competing interests

The authors declare that they have no competing interests.

## Authors' contributions

ML is the principal investigator. ML, HT and IS participated in the design of the study. ET, AN and JN have designed and built the data base from which the material was collected and contributed in the writing of the manuscript. HT and SS contributed in drafting, writing, and editing the manuscript. All authors read and approved the final manuscript.
